# Risk factors and bacterial spectrum postoperative infections after esophageal tumor surgery in patients aged ≥60 years

**DOI:** 10.3389/fonc.2025.1538529

**Published:** 2025-05-28

**Authors:** Dan Li, Mingzhu Lin, Huawei Gu

**Affiliations:** Henan Medical Key Laboratory of Precise Prevention and Treatment of Esophageal Cancer, Anyang Tumor Hospital, The Affiliated Anyang Tumor Hospital of Henan University of Science and Technology, Anyang, China

**Keywords:** patients, esophageal tumor, esophageal tumor surgery (ETS), postoperative infections (PI), risk factors

## Abstract

**Purpose:**

Patients undergoing esophageal tumor surgery (ETS) are at increased risk of postoperative infections (PI), particularly those aged ≥60 years. To improve perioperative safety and outcomes in this population, this study aimed to investigate the bacterial spectrum and identify risk factors associated with PI following ETS.

**Methods:**

A total of 747 patients who underwent radical esophagectomy between January and December 2021 were included in this retrospective analysis. Clinical data, including demographic characteristics, comorbidities, surgery-related variables, and laboratory indicators, were collected and analyzed.

**Results:**

Preoperative and intraoperative risk factors for PI were evaluated using univariate and multivariate logistic regression analyses. The overall incidence of PI was 29.6% (221/747). Smoking, prolonged surgical duration, and elevated postoperative blood glucose levels were identified as independent risk factors for postoperative infection.

**Conclusion:**

Elderly patients undergoing ETS are at considerable risk for PI, particularly those with modifiable risk factors such as smoking and hyperglycemia. Identification of high-risk individuals and implementation of targeted preventive strategies may reduce the incidence of postoperative infections and improve surgical outcomes.

## Background

Postoperative infection (PI) is among the most common complications following surgery (1). PI can occur in patients of any age, with its incidence significantly influenced by patient-related risk factors and the type of surgical procedure (2). Complete surgical resection remains the cornerstone of treatment for patients with localized esophageal thoracic squamous carcinoma (ETS) (3). In addition, neoadjuvant and adjuvant chemotherapy or chemoradiotherapy can improve postoperative prognosis in patients with advanced localized ETS (4). Due to immunosuppression, prolonged operative duration, and extensive surgical trauma, patients undergoing esophageal surgery are particularly susceptible to infections, especially respiratory tract infections, as esophageal procedures carry inherent contamination risks (5). Considerable attention has been directed toward PI as a critical postoperative complication in surgical patients.

Recent studies have highlighted the significance of PI of patients on cancer prognosis (6, 7). Many patients present with comorbidities such as hypertension and hyperglycemia, which reduce tolerance to surgical trauma. A proportion of PIs can be prevented through the implementation of evidence-based preventive strategies, among which the identification of sensitive risk factors serves as a cost-effective measure (8).

Guangyuan et al. (9) identified multiple risk factors contributing to postoperative pulmonary infection in patients undergoing minimally invasive esophagectomy. Clinical staff should recognize these factors promptly and implement early prevention and intervention to reduce the incidence of pulmonary infection and improve patient outcomes. Dindo D et al. further reported that smoking index, pleural adhesions, and prolonged operation duration are associated with an increased risk of pulmonary infection following esophagectomy (10). However, limited evidence exists regarding infectious complications and their associated risk factors in patients aged 60 years and older undergoing ETS. The present study aimed to identify key risk factors associated with postoperative infectious complications in this population and to investigate trends in bacterial resistance.

## Materials and methods

### Study design and samples

All patients aged 60 years or older who were diagnosed with esophageal tumors and underwent surgery at Anyang Tumor Hospital between January 2021 and December 2021 were evaluated for inclusion. No universally accepted definition for “elderly” exists. Therefore, patients aged ≥60 years were selected for this study. Detailed clinical data were retrospectively extracted from electronic medical records by trained investigators. A total of 747 patients were screened, including 465 men (62.25%), with an age range of 60 to 87 years (mean: 69.05 ± 5.02 years). Patients were excluded based on the following criteria: presence of infection before admission, use of antimicrobial agents before admission, severe hepatic, renal, or neurological disorders, immune system dysfunction, pregnancy or lactation, diagnosis of another malignancy, or having undergone combined surgical procedures.

Microbial identification and antibiotic susceptibility testing were conducted using the fully automated MicroScan WalkAway plus 96/40 system (Beckman Coulter, China). Antimicrobial susceptibility to additional antibiotic classes was assessed using the standard disk diffusion method on Mueller-Hinton agar plates in accordance with Clinical and Laboratory Standards Institute (CLSI) guidelines. A total of 25 potential risk factors were recorded for each patient and subjected to statistical analysis to identify factors associated with PI.

### Definitions

PI in this study was defined based on the presence of one or more of the following criteria: (1) clinical manifestations consistent with respiratory tract infection; (2) positive bacterial cultures from sputum, blood, or other sterile body fluids; (3) physician-diagnosed infection based on signs such as erythema, tenderness, incision swelling, fever, or elevated white blood cell (WBC) count; and (4) presence of purulent discharge from the surgical incision or drainage site.

### Statistical analysis

All data analyses were conducted utilizing SPSS version 23.0. Categorical variables were expressed as percentages. all as p < 0.05 was deemed to indicate statistical significance. Univariate and multivariate logistic regression analyses were performed to identify PI-associated variables.

## Results

### The distribution of pathogens in nosocomial infection with esophageal tumor patients

A total of 747 patients who met the inclusion criteria were enrolled, comprising 465 males (62.2%) and 282 females (37.7%), with a mean age of 69.04 years. PI occurred in 221 patients, representing an overall incidence of 29.6%. A total of 197 pathogenic strains were isolated from infected cases. Among these, 178 strains (90.36%) were Gram-negative bacteria, 4 strains (2.03%) were Gram-positive bacteria and 19 strains (9.64%) were fungi (**Table 1**).

**Table 1:** Bacterial spectrum for PI.

Pathogen	Number (%)
Klebsiella	58 (29.44%)
Pseudomonas	22 (11.17%)
Enterobacter	21 (10.66%)
Fungus	19 (9.64%)
Stenotrophomonas	13 (6.59%)
Acinetobacter	13 (6.59%)
Escherichia	12 (6.09%)
Serratia	7 (3.55%)
Citrobacter	6 (3.04%)
Proteus	6 (3.04%)
Other	20 (10.15%)

Others pathogens includes Staphylococcus, Enterococcus, Burkholderia, Anaerobion.

### The antimicrobial resistance trends in nosocomial infection among ETS patients

Antimicrobial resistance patterns of Gram-negative pathogens isolated from postoperative infections are summarized in **Table 2**. Among the isolated *Klebsiella pneumoniae* strains, the highest resistance was observed to polymyxin B, followed by ceftazidime, cefoperazone/sulbactam, gentamicin, and amikacin. Notably, the resistance rates of Enterobacter and Acinetobacter to polymyxin B were 92.86% and 90.00%, respectively. High resistance was also detected against ceftazidime (92.86% and 80.00%), cefoperazone/sulbactam (85.00% and 80.00%), and gentamicin (64.29% and 60.00%).

**Table 2:** Antibiotic resistance prevalence rates of major Gram-negative strains.

Antibacterials	Klebsiella (n=58)	Enterobacter (n=21)	Acinetobacter (n=13)	Pseudomonas (n=22)
gentamicin	17 (29.31)	4 (19.05)	6 (46.15)	1 (4.54)
ceftazidime	19 (32.76)	5 (23.81)	5 (38.46)	1 (4.54)
amikacin	16 (27.59)	2 (9.52)	5 (38.46)	1 (4.54)
Polymyxin B	21 (36.21)	7 (33.33)	7 (53.85)	6 (27.27)
Cefoperazone/sulbactam	17 (29.31)	5 (23.81)	5 (38.46)	3 (13.64)

Values are presented as n (%).

Among the 747 patients, 221 (29.6%) developed PI with specific site distribution as follows: 176 cases of pulmonary infection (23.6%), 14 cases of incisional infection (1.8%), 29 cases of anastomotic leakage/fistula-related infection (3.9%), and 9 cases involving other sites (1.2%) (**Table 3**). Among these patients, 432 were classified as stage I–II and 324 as stage III–IV based on tumor pathology. Pulmonary infections were the most common type of PI, while incisional and other site infections showed markedly lower incidence rates.

**Table 3:** Incidence of site infection relative to TNM grade.

TNM grade	Total No. (%)	Site infection, No. of patients (%)					P^a^
		Lung	Incision	Leakage/fistula	Other sites	Total	
I-II (n=423)	423 (56.6)	95 (12.7)	6 (0.8)	18 (2.4)	1 (0.13)	120 (16.1)	0.464
III-IV (n=324)	324 (43.4)	81 (10.8)	8 (1.07)	11 (1.5)	8 (1.1)	108 (14.5)	0.390
Total	747 (100)	176 (23.6)	14 (1.8)	29 (3.9)	9 (1.2)	228 (30.5)	

a Total number of surgical site infections was compared using the Fisher exact test.

### Univariate analysis of PI risk factors in esophageal tumor patients

The study included 747 patients, 465 men and 282 women, with a mean age of 66.56 ± 7.32 years (range: 60–87 years). No significant difference in age was observed between male and female patients. Demographic characteristics, surgery-related variables, and perioperative clinical data are summarized in **Table 4**. The most frequently performed surgical procedure was transthoracic esophagectomy (479 cases, 64.12%), followed by thoracoscopy and/or laparoscopy-assisted esophagectomy (268 cases, 35.88%). Univariate analysis identified several variables significantly associated with the occurrence of postoperative infection. Higher infection rates were observed in relation to sex (*P* < 0.05), smoking history (*P* < 0.01), hemoglobin level (*P* < 0.05), duration of surgery (*P* < 0.05), postoperative WBC count (*P* < 0.05), presence of pulmonary disease (*P* < 0.05), and postoperative blood glucose levels (*P* < 0.05).

**Table 4:** Results of univariate analysis of demographic characteristics, surgery-related Variables, and perioperative data.

Variable	All enrolled patients	PI (-)	PI (+)	OR (95% CI)	P
Age (years)	≥60 (n=747)	66.59 ± 7.34 (349)	66.56 ± 7.32 (398)	1.006 (0.750-1.350)	0.11^a^
Sex	Male (n=465)	204	261	1.354 (1.006-1.82)	0.045^b^
	Female (n=282)	145	137		
Smoking	Yes (n=297)	117	180	1.637 (1.216-2.204)	0.001^b^
	No (n=450)	232	218		
Drinking	Yes (n=123)	53	70	1.023 (0.694-1.508)	0.907^b^
	No (n=624)	296	382		
Diabetes mellitus	Yes (n=102)	41	61	1.360 (0.889-2.080)	0.155^b^
	No (n=645)	308	337		
Hypertension	Yes (n=260)	125	135	0.920 (0.680-1.244)	0.587^b^
	No (n=487)	224	263		
Hyperlipidemia	Yes (n=36)	16	20	1.101 (0.561-2.160)	0.779^b^
	No (n=711)	333	378		
Previous radiotherapy	Yes (n=16)	10	6	0.519 (0.187-1.443)	0.201^b^
	No (n=731)	339	392		
Previous chemotherapy	Yes (n=165)	77	88	1.003 (0.709-1.418)	0.030^b^
	No (n=582)	272	310		
Preoperative hospital stays (days)	>8 (n=389)	172	217	1.234 (0.925-1.646)	0.153^b^
	≤ 8 (n=358)	177	181		
WBC count	Normal (n=712)	332	380	1.145 (0.366-3.583)	0.968^b^
	High (n=12)	6	6		
	Low (n=23)	11	12		
Albumin count	Normal (n=708)	331	377	0.976 (0.511-1.864)	0.942^b^
	Low (n=39)	18	21		
Tumor recurrence	Yes (n=5)	3	2	0.582 (0.097-3.506)	0.550^b^
	No (n=742)	346	396		
TNM grade	I–II (n=423)	199	224	0.970 (0.726-1.297)	0.839^b^
	III–IV (n=324)	150	174		
Tumor site	upper-thoracic (n=126)	57	69	1.130 (0.762-1.678)	0.506^b^
	mid-thoracic (n=468)	226	242		
	Lower-thoracic (n=153)	66	87		
Hemoglobin	Normal (n=246)	113	133	1.177 (0.073-19.031)	0.025^b^
	High (n=2)	1	1		
	Low (n=499)	278	314		
Surgical duration (h)	>4 (n=345)	146	199	1.390 (1.041-1.857)	0.026^b^
	≤4 (n=402)	203	199		
Surgical method	Endoscopic (n=479)	221	258	1.067 (0.791-1.440)	0.670^b^
	Thoracotomy (n=268)	128	140		
Post-operative fever	>38.5 (n=11)	3	8	2.366 (0.623-8.988)	0.139^b^
	≤38.5 (n=736)	346	390		
Post-operative WBC count	Normal (n=189)	100	89	0.704 (0.505-0.981)	0.038^b^
	High (n=548)	242	306		
	Low (n=10)	7	3		
Intra-operative blood loss (ml)	<200 (n=599)	277	322	1.079 (0.743-1.569)	0.806^b^
	200-400 (n=135)	65	70		
	>400 (n=13)	7	6		
Intraoperative transfusion	<200 (n=741)	348	393	0.282 (0.031-2.538)	0.312^b^
	200-400 (n=5)	1	4		
	>400 (n=1)	0	1		
Pulmonary disease	Yes (n=102)	38	64	1.568 (1.020-2.411)	0.039^b^
	No (n=645)	311	334		
Postoperative blood glucose	Yes (n=576)	255	321	1.537 (1.090-2.166)	0.014^b^
	No (n=171)	94	77		
Hospitalization times	>1 (n=323)	158	165	0.856 (0.640-1.144)	0.294^b^
	1 (n=424)	191	233		

(a) p-value by Student’s t-test. (b) p-value by Chi-square test.

### Multivariate logistic regression analysis of risk factors of PI in ETS patients

**Figure 1:**
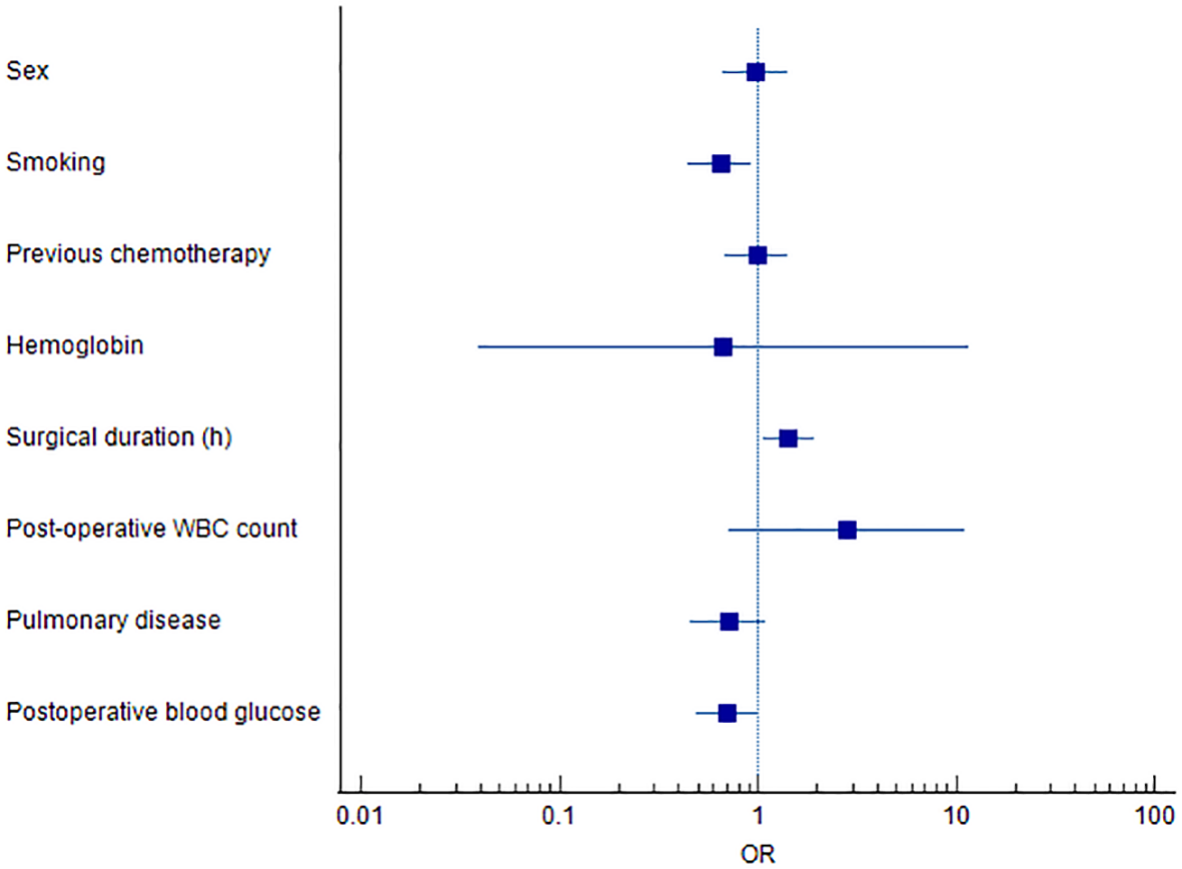
Forest plot of independent risk factors associated with postoperative infection in ETS patients.

A multivariate logistic regression model was constructed using variables identified as significant in the univariate analysis, including sex, smoking history, prior chemotherapy, hemoglobin level, surgical duration, postoperative white blood cell count, pulmonary disease, and postoperative blood glucose. The analysis identified smoking, prolonged surgical duration, and elevated postoperative blood glucose as independent risk factors significantly associated with the development of PI in elderly patients undergoing ETS (*P* < 0.05) (**Table 5**, **Figure 1**).

**Table 5:** Risk factors for PI identified by multivariate logistic regression analysis with esophageal tumor patients.

Variable		PI (-)	PI (+)	OR (95% CI)	P
Sex	Male (n=465)	204	261	0.985 (0.676-1.435)	0.985
	Female (n=282)	145	137		
Smoking	Yes (n=297)	117	180	0.650 (0.448-0.941)	0.023
	No (n=450)	232	218		
Previous chemotherapy	Yes (n=165)	77	88	0.990 (0.689-1.421)	0.955
	No (n=582)	272	310		
Hemoglobin	Normal (n=246)	113	133	0.672 (0.039-11.538)	0.806
	High (n=2)	1	1		
	Low (n=499)	278	314		
Surgical duration (h)	>4 (n=345)	146	199	1.432 (1.061-1.932)	0.019
	≤4 (n=402)	203	199		
Post-operative WBC count	Normal (n=189)	100	89	2.822 (0.711-11.200)	0.140
	High (n=548)	242	306		
	Low (n=10)	7	3		
Pulmonary disease	Yes (n=102)	38	64	0.712 (0.455-1.112)	0.135
	No (n=645)	311	334		
Postoperative blood glucose	Yes (n=576)	255	321	0.697 (0.490-0.992)	0.045
	No (n=171)	94	77		

### Effect of infection status on Kaplan-Meier survival analysis

**Figure 2:**
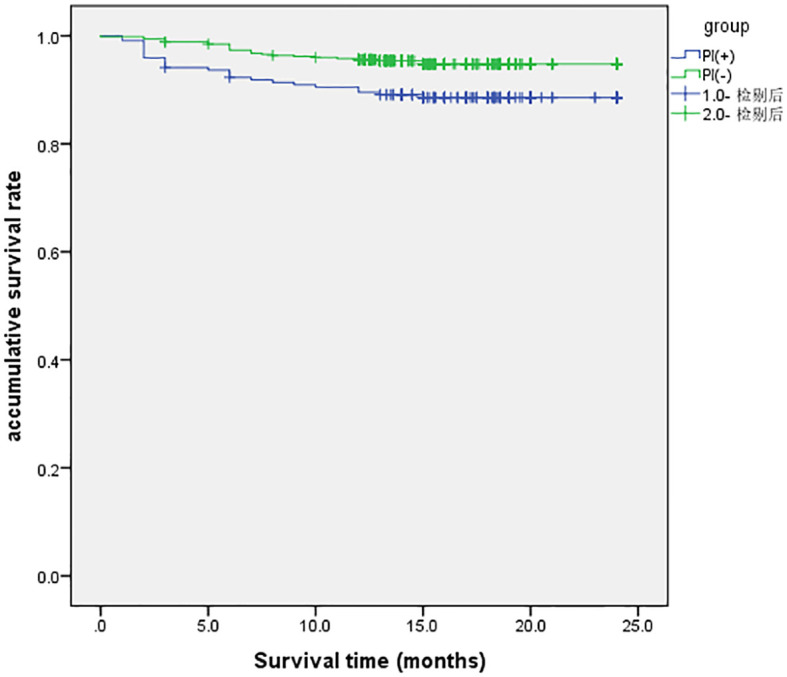
Kaplan-Meier survival analysis of between PI and non-PI patients with esophageal tumors.

Patients were stratified into two groups based on PI status: those who developed PI and those who did not (**Figure 2**). After excluding 7 patients lost to follow-up, survival analysis was conducted on 740 patients. The two-year survival rate was 95.0% in the non-PI group, compared to 88.6% in the PI group. Kaplan–Meier analysis revealed a statistically significant difference in survival between the two groups (*P*<.05).

## Discussion

The present study aimed to identify risk factors associated with nosocomial PI and to characterize the bacterial spectrum in patients aged ≥60 years undergoing ETS. The overall incidence of PI in this cohort was 29.6%. Gram-negative bacteria accounted for most identified pathogens, and the predominant pattern of polymicrobial infections involved a combination of Gram-negative bacteria and fungi. *Klebsiella pneumoniae* was the most frequently isolated pathogen, followed by *Pseudomonas aeruginosa*, *Enterobacter cloacae*, and *Candida albicans*, each representing more than 10% of the documented isolates. These findings align with previous studies, which reported that *Haemophilus influenzae*, *Klebsiella pneumoniae*, *Pseudomonas aeruginosa*, and *Enterobacter cloacae* were detected in approximately 70% of postoperative pulmonary infections. In addition, *Staphylococcus aureus* has also been frequently implicated in such cases (11, 12).

Our previous study demonstrated that a prolonged preoperative hospital stay increased the risk of postoperative infection in esophageal tumor patients (*p* < 0.05) (13). A likely explanation is that extended hospitalization facilitates colonization by multisite pathogens and elevates the risk of cross-infection. In the current study, smoking, surgical duration, and postoperative blood glucose levels (**Figure 1**) were identified as significant risk factors for PI following ETS. Notably, surgical duration and postoperative blood glucose are potentially modifiable, offering actionable targets for clinical intervention. Meanwhile, Tobacco smoking is also a risk factor for several adverse post-operative outcomes (14). Smoking impairs pulmonary function through mechanisms such as ciliary damage, impaired mucociliary clearance, and increased mucus production (15), thereby promoting pathogen colonization and elevating the risk of postoperative pneumonia (16). These findings are consistent with previous literature, which has emphasized smoking as a significant contributor to postoperative respiratory complications. Akutsu et al. (17) highlighted the benefits of perioperative management strategies, including smoking cessation and preoperative respiratory rehabilitation, in reducing postoperative pneumonia following esophagectomy. Such interventions enable smokers to quit before surgery, facilitate the practice of coughing and deep-breathing exercises, and promote adherence to nutritional and physical rehabilitation programs (18, 19).

The present study confirms that prolonged surgical duration serves as an independent predictor of postoperative pulmonary complications. Previous guidelines from the American College of Physicians have similarly highlighted that procedures lasting between 3 to 4 hours are significantly associated with an increased risk of perioperative pulmonary events (20, 21). In our study population, patients undergoing esophageal tumor surgery with an operative time exceeding 4 hours showed a markedly elevated risk of PI (OR value 1.43). The resistance patterns are concerning. Among various risk factors, surgical duration directly influences the length of exposure of the surgical wound to environmental pathogens (22). Prolonged operative time increases the risk of surgical site infection due to extended wound exposure to ambient air (23). When prolonged surgical duration is anticipated, adjustments to preoperative prophylactic antibiotic protocols should be considered, and intraoperative manipulation should be performed with care to minimize trauma to surrounding structures (24). Although the duration of surgery is inherently influenced by the procedure type and patient complexity, optimized preoperative planning and enhanced intraoperative efficiency may prevent unnecessary prolongation, ultimately reducing the risk of PI.

Naoko Ito et al. reported that hyperglycemia on postoperative day 3 was a significant predictor of postoperative infections following highly invasive esophageal cancer surgery (25). Dronge et al. demonstrated that adequate preoperative glycemic control (HbA1c levels <7.0%) was associated with a reduced incidence of postoperative infectious complications across a range of major noncardiac surgical procedures (26).

The incidence of postoperative infection was notably higher among patients with diabetes, and several pathophysiological mechanisms may contribute to this increased risk. In individuals with diabetes, persistent hyperglycemia leads to elevated plasma osmotic pressure, which impairs leukocyte chemotaxis, phagocytosis, and adhesion. These impairments weaken the host immune defense against pathogens, increasing susceptibility to infection (27). Diabetic patients often exhibit significantly higher HbA1c levels compared to non-diabetic individuals. Elevated HbA1c shifts the oxyhemoglobin dissociation curve to the left, enhancing oxygen-binding affinity but impairing oxygen release to tissues. This impairs gas exchange and reduces oxygen delivery to pulmonary capillary beds, exacerbating the risk of pulmonary infections (28). Surgical trauma induces a stress response contributing to peripheral insulin resistance and inhibiting pancreatic β-cell insulin secretion. Reduced insulin activity results in perioperative hyperglycemia, suppressed protein synthesis, enhanced protein catabolism, and an increased risk of infection (29). Vitamin A metabolism is impaired in diabetic patients due to reduced hepatic conversion, leading to diminished respiratory mucosal protection. This impairment predisposes lung tissue to infection (30). These findings highlight the critical importance of perioperative glycemic control in diabetic patients. To reduce the risk of postoperative infection, blood glucose should be carefully managed and maintained within the target range of 7.8–11.1 mmol/L during both intraoperative and early postoperative periods (31).

The findings of this study should be considered when formulating targeted intervention strategies to reduce the risk of postoperative infection. Further research is warranted, particularly studies exploring the utility of preoperative sputum or fecal screening, the effectiveness of perioperative antimicrobial prophylaxis, and optimal management strategies for PI in esophagectomy patients.

In conclusion, this retrospective study suggests that PI remains a common complication following esophagectomy, despite recent improvements in perioperative care. Gram-negative bacteria were identified as the predominant pathogens in patients aged ≥60 years. Smoking, prolonged operative duration, and postoperative hyperglycemia were significantly associated with an increased risk of infection, potentially contributing to a higher incidence of postoperative complications. However, due to the inherent limitations of the retrospective study design, causal relationships cannot be definitively established.

## Data Availability

The original contributions presented in the study are included in the article/supplementary material. Further inquiries can be directed to the corresponding author.
